# Machine learning corroborates subjective ratings of walking and balance difficulty in multiple sclerosis

**DOI:** 10.3389/frai.2022.952312

**Published:** 2022-09-29

**Authors:** Wenting Hu, Owen Combden, Xianta Jiang, Syamala Buragadda, Caitlin J. Newell, Maria C. Williams, Amber L. Critch, Michelle Ploughman

**Affiliations:** ^1^Ubiquitous Computing and Machine Learning Research Lab, Department of Computer Science, Memorial University of Newfoundland, St. John's, NL, Canada; ^2^Recovery and Performance Laboratory, Faculty of Medicine, Memorial University of Newfoundland, St. John's, NL, Canada

**Keywords:** artificial intelligence, gait analysis, machine learning, multiple sclerosis, walkway, rehabilitation

## Abstract

Machine learning can discern meaningful information from large datasets. Applying machine learning techniques to raw sensor data from instrumented walkways could automatically detect subtle changes in walking and balance. Multiple sclerosis (MS) is a neurological disorder in which patients report varying degrees of walking and balance disruption. This study aimed to determine whether machine learning applied to walkway sensor data could classify severity of self-reported symptoms in MS patients. Ambulatory people with MS (*n* = 107) were asked to rate the severity of their walking and balance difficulties, from 1-No problems to 5-Extreme problems, using the MS-Impact Scale-29. Those who scored less than 3 (moderately) were assigned to the “mild” group (*n* = 35), and those scoring higher were in the “moderate” group (*n* = 72). Three machine learning algorithms were applied to classify the “mild” group from the “moderate” group. The classification achieved 78% accuracy, a precision of 85%, a recall of 90%, and an F1 score of 87% for distinguishing those people reporting mild from moderate walking and balance difficulty. This study demonstrates that machine learning models can reliably be applied to instrumented walkway data and distinguish severity of self-reported impairment in people with MS.

## Introduction

Machine learning is a valuable tool for dealing with enormous amount of data and discovering new knowledge from that data, which has been widely employed in gait detection and analysis from various types of sensors, including videos, inertial measurement unit (IMU), surface electromyography, and insoles (Khera and Kumar, 2020; Saboor et al., 2020; Iosa et al., 2021; Seo et al., 2021). Instrumented walkways, widely used in field of gait biomechanics, sensitively map the spatiotemporal profile of gait. The walkway consists of a dense matrix of embedded sensors to capture walking data from gait. With the sophisticated software package destinated to the walkway system, regular spatiotemporal gait parameters can be estimated and visualized for assisting in various neural-degenerative disease evaluation and diagnosis, such as stroke and multiple sclerosis (Buckley et al., 2018; van de Port et al., 2020; Khera and Kumar, 2022).

MS is a degenerative central nervous system disease (Reich et al., 2018) with a prevalence of 35.9 per 100,000 people worldwide (MSIF, 2020). Patients with MS experience limb weakness, sensory loss, and foot drop which sometimes go undetected until clearly observed by others (Socie et al., 2013; Reich et al., 2018). Patients may sense subtle changes in their walking and balance before these symptoms can be detected by clinicians (Kirkland et al., 2018, 2020). Machine learning methods have been successfully applied to estimate gait parameters from wearables in multiple sclerosis. For example, McGinnis et al. estimated gait speed of MS patients from an array of accelerometers attached to different locations on the body (McGinnis et al., 2017). Our previous published work (Hu et al., 2022) applied machine learning to successfully detect MS patients from healthy volunteers using novel features combined with standard features derived from the raw walkway sensor data. Also based on instrumented walkway raw data, Trentzsch et al. explored different machine learning algorithms (Gaussian Naive Bayes, Decision Tree, k-Nearest Neighbor, and Support Vector Machines) to distinguish mild and moderate MS levels defined by Expanded Disability Status Scale (EDSS) (Trentzsch et al., 2021). However, a recent cross-sectional study showed that spatiotemporal gait measures have great variability within homologous EDSS categories suggesting that EDSS insensitive to subtle differences (Zanotto et al., 2022). In this study, we explored whether machine learning could be applied to raw walkway data to discern the severity of subjective walking difficulties in people with MS (or other neurological disorders). We focused on the patients' perspective because recent evidence suggests that MS has a long prodromal period (up to 5 years) when gait and balance symptoms can go undetected by clinicians (Wijnands et al., 2019).

We grouped patients by their self-reported walking and balance problems using questions in the MS Impact Scale−29 (Phillips et al., 2014) and applied machine learning to gait features derived from the walkway sensors (Hu et al., 2022). It was hypothesized that machine learning models could effectively distinguish the targets.

## Materials and methods

### Data collection and experimental design

Following approval by the Institutional Health Research Ethics Board (HREB # 2015.103), raw walkway sensor data were collected from the Health Innovation Team in MS (HITMS) project, which studies the health of people with MS in Newfoundland and Labrador, Canada (Chaves et al., 2019; Galloway et al., 2019). Each patient walked across the instrumented walkway (Zeno Walkway, Protokinetics Haverton PA) which measured 90 × 420 cm with or without a walking assistive device (Severini et al., 2017). Spatial data were collected from pressure sensors which represented in x, y coordinates and had an active area of 1.27 × 1.27 cm, 1 cm apart from each other. By calculating Euclidean distance between sensors, the value of the gait features can be obtained^1^. Participants performed two different walking tests. Firstly, they walked four times across the walkway at their self-selected comfortable speed. After a rest, the participants performed a dual task walking test at their self-selected speed for four times. During this dual task walking, the participant was asked to walk while subtracting 7 from a preceding number beginning with 100, and speaking aloud the results (Kirkland et al., 2015; Chen et al., 2020; Hu et al., 2022).

Expanded Disability Status Scale (EDSS) rating by the MS neurologist were extracted from health records. Those patients with EDSS scores of higher than 6.5, who could not walk, were excluded. Thus, data from 107 patients were included. Their EDSS scores range from 0 to 6.5, and their average EDSS score was 2.11 ± 1.89.

The replies to a subset of eight MSIS-29 questions on how patients felt about their walking, balance, and movement (Table 1) were used to determine the self-reported rating of walking problems. Patients who scored less than 3 (answering “not at all” or “a little” to the MSIS-29) were considered to have mild walking and balance problems. The remaining patients who scored 3 (“moderate”), 4 (“quite a bit”) and 5 (“extreme”) were considered moderate. There were five different levels of severity, but since there were too few participants in some of the groups, we decided to split the groups into moderate and mild^2^.

**Table 1:** Patient demographic data and MSIS-29 scores.

**Characteristics**	**Moderate** **(mean ± SD)**	**Mild** **(mean ± SD)**
Gender	48 Women 24 Men	27 Women 8 Men
EDSS Score	2.73 ± 2.04	0.81 ± 1.54
Age	48.40 ± 9.95	46.82 ± 10.35
MSIS-29-Q4 Problems with your balance?	2.99 ± 0.94	1.40 ± 1.37
MSIS-29-Q5 Difficulties moving about indoors?	2.14 ± 1.01	1.10 ± 0.86
MSIS-29-Q6 Being clumsy?	2.76 ± 1.01	1.35 ± 1.22
MSIS-29-Q7 Stiffness?	2.86 ± 1.15	1.39 ± 1.24
MSIS-29-Q8 Heavy arms and/or legs?	2.90 ± 1.14	1.22 ± 1.42
MSIS-29-Q9 Tremor of your arms or legs?	2.17 ± 1.17	1.07 ± 0.89
MSIS-29-Q10 Spasms in your limbs?	2.29 ± 1.25	1.10 ± 0.97
MSIS-29-Q11 Your body not doing what you want it to do?	2.39 ± 1.21	1.20 ± 1.00

### Gait features

A set of gait features was extracted from the raw sensor data based on footprints from each pass. These included 21 features, foot type/length/width/area, unsigned toe angle, step/stride length, step/stride width, base width, step/stride time, step/stride velocity, single/double support time, stance time, toe direction, hull area, base of support (BOS) area, line of progression (LOP) deviation angle. The details regarding each parameter and how they were extracted from the walkway sensor data were described previously (Hu et al., 2022).

### Machine learning process

**Figure 1:**
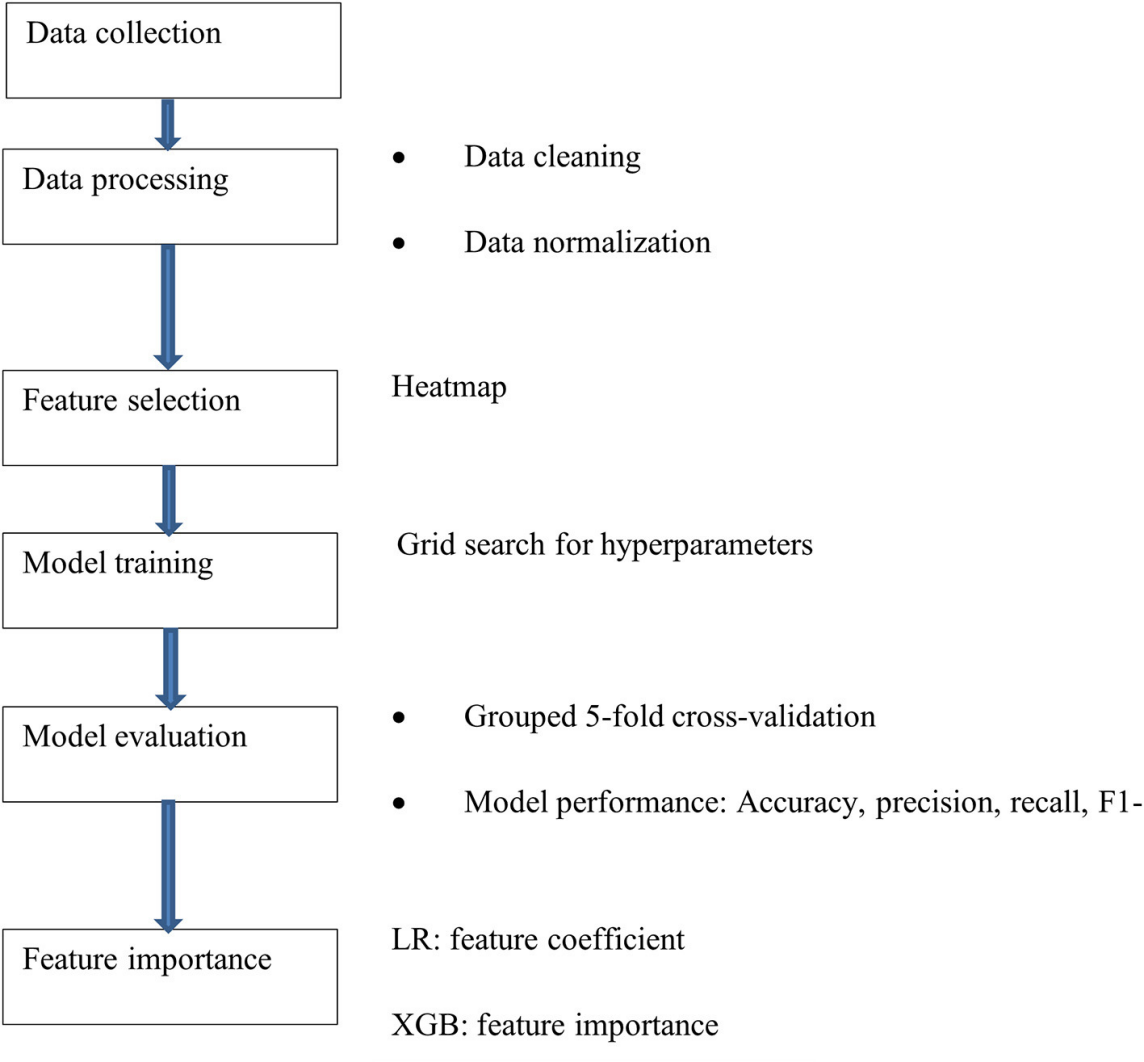
Machine learning process.

The machine learning process is illustrated in Figure 1. Data were cleaned negative values from time features were excluded, incorrectly clustered data were checked and eliminated from the dataset. The remaining data were scaled to exhibit zero mean and unit variance. Feature selection were completed using ANOVA-SVM based on previous work (Hu et al., 2022). However, in this paper, we used the adaptive synthetic sampling approach (ADASYN) for data balancing and managing different hyperparameters for each model. The importance of each feature to model prediction was also calculated.

#### Data balancing

Imbalanced data means that the number of samples belonging to each class in the problem is not evenly distributed. For example, if 90% of the data belong to the same class, models will reach 90% accuracy by classifying all the data into the same class, and such a model is biased. In our case, the data of mild/moderate patients had a ratio of 1:3. Therefore, data balancing was performed (Menardi and Torelli, 2014). The ADASYN (He et al., 2008) was used to synthesize new samples close to minority class samples that are harder to learn, according to the possibility of the correctly classified minority sample's neighbor (K nearest neighbors) (He et al., 2008).

#### Feature importance

Feature importance explains the model results and which features are vital in distinguishing the targets. We evaluated the feature importance through three different types of models, intended to check which features are consistently important between models. The pipeline ANOVA-SVM (Megantara and Ahmad, 2021) calculates the average F-score for the selected features. Linear models calculate the coefficient of each feature to determine feature importance. Feature importance for tree-based models is calculated based on how the nodes of the tree used in training improve the model results. Then, feature scores are scaled to have their sum equal to one. The higher the score, the higher the importance of the feature. The scores of the features are automatically computed by the provided sciki-learn software (Pedregosa et al., 2011) when training is completed.

#### Machine learning algorithms

Logistic regression (LR) (Cox, 1958), support vector machine (SVM) (Cortes and Vapnik, 1995), and extreme gradient boosting (XGB) (Chen and Guestrin, 2016) were selected, as these three algorithms represent three accepted classification methods representing linear, non-linear, and decision tree-based classifiers.

LR provides a probability for the target class between 0 and 1 to describe the relationship between the input variables and one or more output targets. The input value x is fitted into the logistic function, whereby the weights or coefficient values are adjusted to predict the output value y.

SVM has the capability to work with high-dimensional data. SVM attempts to define a hyperplane boundary in an N-dimensional space, where N is the number of input features. SVM is supposed to determine the optimal plane that best separates the classes.

XGB is a distributed gradient boosting machine learning library. Applied to an ensemble of weak prediction models, the gradient boosting algorithm trains data with these weak learners forces the poor performance learners to learn to increase their prediction score, and finally combines them into one accurate prediction algorithm.

#### Training and evaluation

A standard grid search method was used to choose the best hyperparameters. The hyperparameter options and the optimal hyperparameters for each model described in Table 2.

**Table 2:** Hyperparameter options for each model.

**Algorithms**	**Hyperparameter options**	**Optimal hyperparameters**
LR	“solver”: [“newton-cg”, ”lbfgs”, “liblinear”], “penalty”: [“l1”, “l2”, “elasticnet”], “C”: [100, 10, 1.0, 0.5], “max_iter”: [200, 400, 600]	“solver”: “liblinear” “penalty”: “l2”, “C”: 10, “max_iter”: 200,
SVM	“kernel”: [“poly”, “rbf”, “sigmoid”, “linear”], “C”: [8, 7, 6, 5, 4], “degree”: [0, 1, 2]	“kernel”: “rbf”, “C”: 8, “degree”: 0,
XGB	“max_depth”: [3, 4, 5, 6], “eta”: [0.05, 0.1, 0.2, 0.3, 0.4], “objective”: [“binary: logistic”, “binary: logitraw”, “binary: hinge”]	“max_depth”: 6, “eta”: 0.05, “objective”: “binary: hinge”

Grouped 5-fold cross-validation strategy was employed to evaluate the model result (Hu et al., 2022). Accuracy, precision, recall, and F1-score were calculated to score the model's effectiveness.

We compared the means of accuracies between the three models using Student's *T*-test, and means of areas under curve (AUC) using Delong test (Delong et al., 1988; Sun and Xu, 2014).

## Experimental results

### Feature selection based on feature correlations

**Figure 2:**
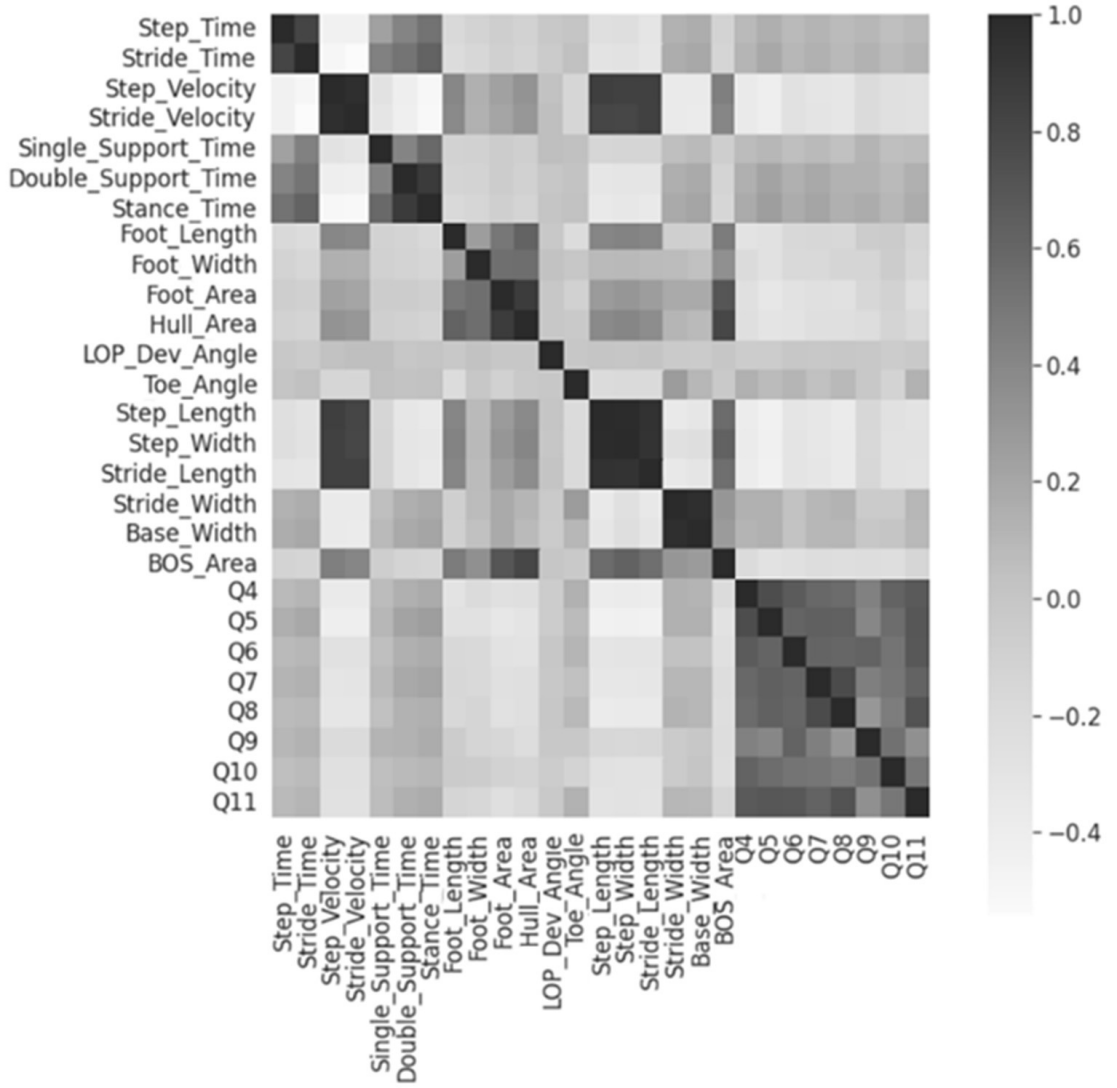
Heatmap for numerical feature correlations. Heatmap regions that are increasingly dark show areas of higher correlations. Q4-Q11 represents the MSIS-29 questions 4 to 11.

The 19 numerical feature correlations were visualized using a heatmap, as shown in Figure 2. None of the features were significantly correlated with the MSIS-29 questions. However, a strong positive correlation was discovered between the “step” and “stride” parameters and the base/stride width. Therefore, 3 features stride time, stride velocity, and base width were excluded from further analysis.

ANOVA-SVM then determined that all remaining 16 features contributed to model prediction; respectively they were step time/velocity, single/double support time, stance time, foot length/width/area, hull area, LOP deviation angle, toe angle unsigned, step/stride length, step/stride width, BOS area. Foot type and toe direction were then reintroduced for further analysis.

### Feature importance

The average F-score values of features from ANOVA-SVM, the absolute value of feature coefficient of features from LR, feature importance from XGB are presented below.

**Figure 3:**
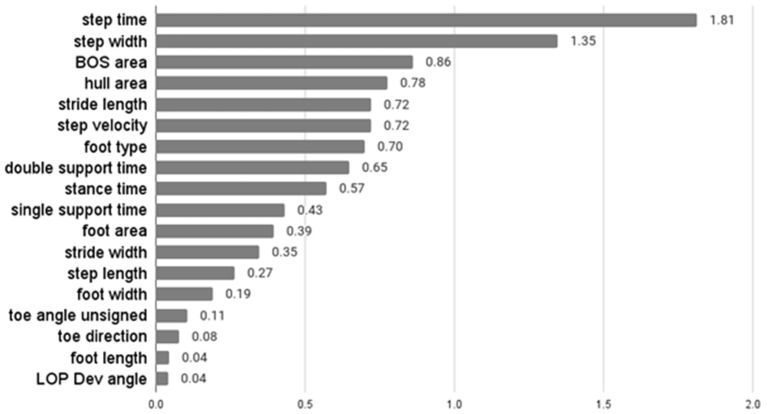
Absolute values of feature coefficient of LR.

**Figure 4:**
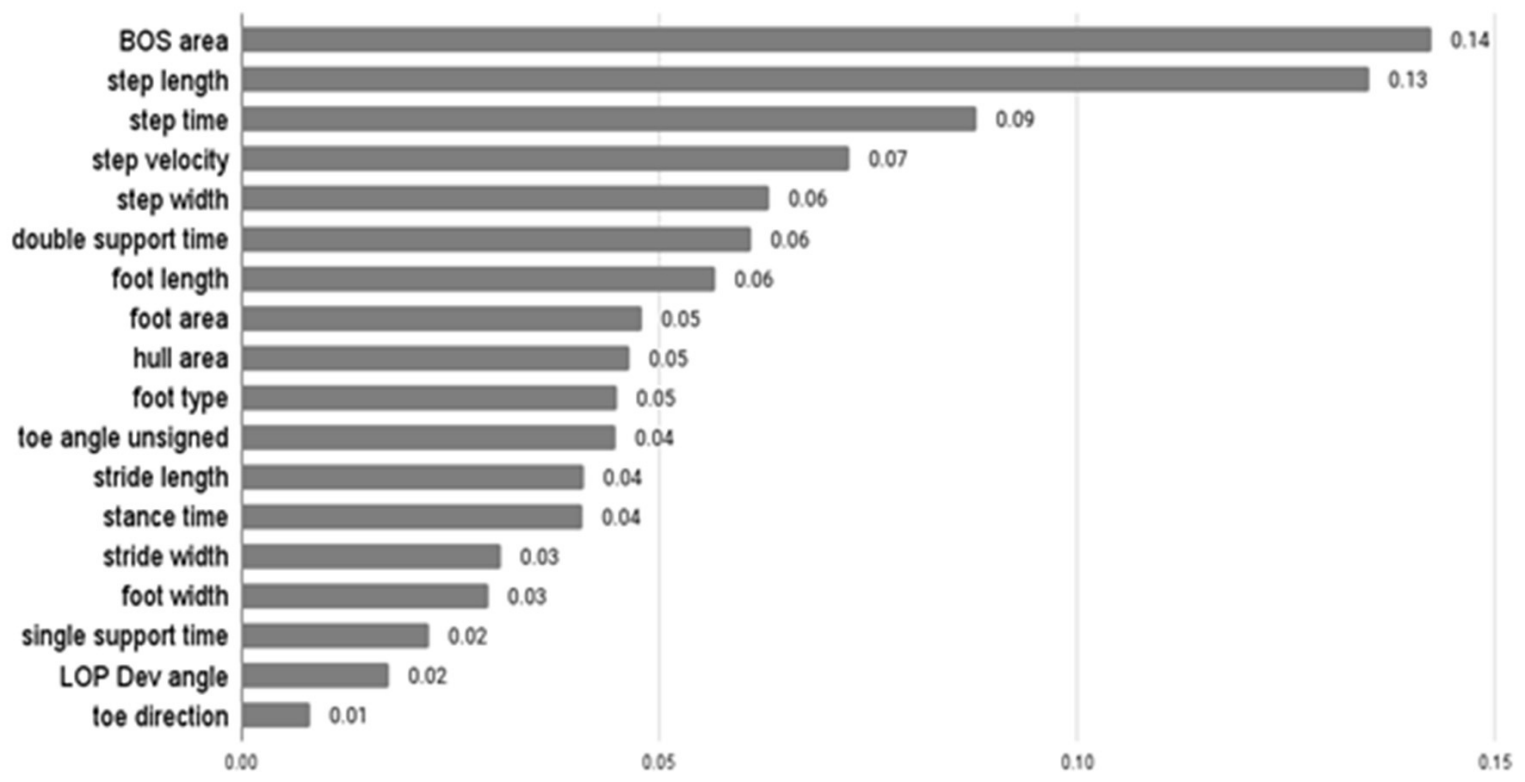
Feature importance of XGB.

Table 3 indicates that step width, step length, BOS area, and hull area were the top four most important features for the classification examined by ANOVA-SVM. Step time was the most important feature of LR, followed by step width and area-related features (Figure 3). For the XGB classifier (Figure 4), BOS area was the most important feature, followed by the step length and step time. Step velocity and step width were considered less important. Among all three types of models, step time and step width were considered important.

**Table 3:** Average F- score for features.

**Features**	**Average F-scores**
Step width	561.98
Step length	504.57
BOS area	416.50
Hull area	402.72
Stride length	354.94
Foot area	339.31
Foot length	261.41
Step velocity	181.54
Foot width	60.07
Toe angle unsigned	46.48
Double support time	39.89
Stance time	35.75
Step time	35.70
LOP Dev angle	10.66
Single support time	4.90
Stride width	1.41

### Prediction results

**Figure 5:**
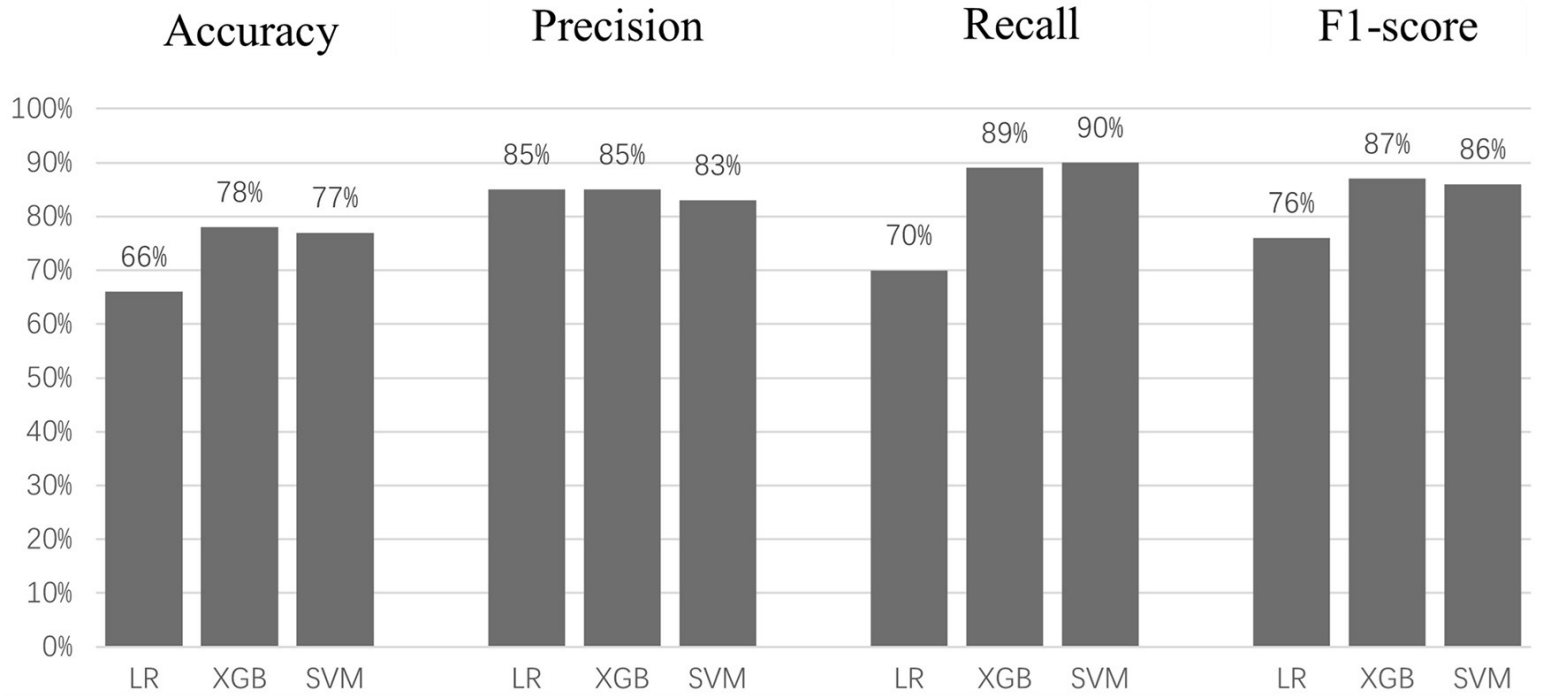
Accuracy, precision, recall, and F1 score for each model.

Figure 5 and Table 4 show the prediction results of three models. The student's *t*-test shows the accuracy of LR (66%) is significantly lower (*p* < 0.005) than those of XGB (78%) and SVM (77%); there is no significant difference between the accuracies of XGB and SVM. Similarly, LR has significantly lower recall (70%) and f1-score (76%) values than those of XGB (recall: 89%, f1-score: 87%) and SVM (recall: 90%, f1-score: 86%), with no significant differences between XGB and SVM. There was no significant difference between the precision values of the three models. However, the Delong test shows there are significant differences between means of AUC of all three models (*p* < 0.0001), with XGB being the best (98.1%), SVM (96%), and LR (79.6%). The reason the AUC values of all three models are higher than model accuracies could be that the optimized decision thresholds were chosen for calculating AUCs (Bradley, 1997).

**Table 4:** Accuracy, precision, recall, and F1 score for each model.

	**LR**	**XGB**	**SVM**
Accuracy	66%	78%	77%
Precision	85%	85%	83%
Recall	70%	89%	90%
F1-score	76%	87%	86%

## Discussion

This study demonstrates that different degrees of walking and balance problems reported by MS patients can be distinguished by the gait features calculated from the raw walkway sensor data using machine learning methods. Although only about 78% accuracy was achieved by XGB, it is comparable to the accuracy reported by Trentzsch et al. (2021) in distinguishing moderate and mild MS divided by EDSS score), where an accuracy of about 64% was achieved by using majority decision among six machine learning models. Furthermore, 85% of the ‘moderate’ group was correctly identified (recall) by the XGB classifier and 90% of predicted ‘moderate’ samples identified by SVM were correct (precision), which are also comparable to the corresponding results reported by Trentzsch et al. (2021).

The most important gait features detected in this work were different than those identified in our previous study when distinguishing MS patients from healthy controls (Hu et al., 2022). The previous work identified stride length as the most important feature; a gait characteristic that strongly distinguished MS patients from healthy controls. In this study, step width, step time and base of support area became important, suggesting that problems with stability are distinguishing characteristics when detecting more subtle gait and balance changes. Patients may adapt to feeling unsteady by widening their feet and walking more slowly to prevent falls (Ploughman et al., 2014; Chen et al., 2020). As reported by Brach et al. (2005), people with either narrow or wide step width were more likely to report a fall, and step width could also be an indicator in identifying patients with potential gait disability. Interestingly, foot area-related features including the base of support area, foot area, and hull area were of high importance both for determining the severity of the patient's condition and distinguishing MS patients from healthy controls as reported by our previous study (Hu et al., 2022), which might hint that the patient's balance might be affected by disease progression (Singh and Kelly, 2009).

Machine learning algorithms have been applied to other neurological diseases to gauge severity of walking and balance problems. For instance, machine learning algorithms applied to vertical ground reaction force data predicted severity levels of Parkinson's disease patients according to the Hoehn & Yahr scale (Hoehn and Yahr, 1967; Zhao et al., 2018). Center of pressure coordinates and trajectories were used as prediction features to rate level of gait impairment in Parkinson's disease and cerebral palsy (Mancinelli et al., 2012; Khera and Kumar, 2022). Importantly, these projects used clinician-reported scores as ground truth; impairments that can be observed by another person. The current work uniquely corroborates the patient's perspective regarding their own walking problems, some of which may not be noticeable by others.

It is worth mentioning the limitations of the present work. We employed only two levels (mild vs. moderate) of self-reported (and combined) gait and balance problems using binary class models. Future research should explore expanded ratings, such as mild, moderate, and severe, or divide self-reported mobility problems based on whether the problem relates to gait or balance. Thus, more novel features would be explored, including pressure distribution of the foot, which could potentially be helpful for classification (Kaya et al., 2022) and accuracy. The data used in this project originates from 107 patients, and although this is a fairly large sample in some types of research, a larger sample size may further increase the model performance. Feature importance determination in this study was limited to model specific methods. In the future, more sophisticated and model-agnostic methods such as *Lorenz-Zonoid method* (Giudici and Raffinetti, 2021) would provide more explainable comparison of the variable importance. Finally, this study included two walking datasets, self-selected speed walking and dual-task walking. Combining the datasets provided a certain level of robustness of the machine learning model, but more walking tests such as fast-paced walking should be included in the future work to exhaustively interrogate the models.

## Conclusion

This study demonstrated that machine learning can be used to classify patients' self-reported disability levels using only the raw data collected from an instrumented walkway system. We achieved 78% accuracy, 85% recall, and 90% precision when classifying mild patients from moderate patients using SVM.

Step time, BOS area and step width contributed most to classifying mild patients from moderate patients suggesting changes in balance.

## Data availability statement

The datasets used and analyzed during the current study will be made available by the corresponding author upon reasonable request.

## Ethics statement

The studies involving human participants were reviewed and approved by Institutional Health Research Ethics Board (HREB No. 2015.103). The patients/participants provided their written informed consent to participate in this study.

## Author contributions

WH: experiment design, data analysis and interpretation, and paper drafting and editing. OC: experiment design and data analysis and interpretation. MP and XJ: research concept design, paper revision, and funding support. CN, MW, SB, and AC: subject recruitment, data collection and extraction, and data cleaning. All authors contributed to the article and approved the submitted version.

## Funding

This work was supported in part by Natural Sciences and Engineering Research Council of Canada (NSERC) Discovery [XJ, Grant No. RGPIN-2020-05525], the Canada Research Chairs Program (MP Grant No. 950-230457), Canada Foundation for Innovation (MP Project No. 33621), Canadian Institutes for Health Research (MP Grant No. 169649).

## Conflict of interest

The authors declare that the research was conducted in the absence of any commercial or financial relationships that could be construed as a potential conflict of interest.

## Publisher's note

All claims expressed in this article are solely those of the authors and do not necessarily represent those of their affiliated organizations, or those of the publisher, the editors and the reviewers. Any product that may be evaluated in this article, or claim that may be made by its manufacturer, is not guaranteed or endorsed by the publisher.

## Footnotes

^1^GAITRite Electronic Walkway Technical Reference (47DevM2).

^2^Multiple Sclerosis Impact Scale (MSIS-29), 2000.
